# Supplementary material for: Beeswax Alcohol and Fermented Black Rice Bran Synergistically Ameliorated Hepatic Injury and Dyslipidemia to Exert Antioxidant and Anti-Inflammatory Activity in Ethanol-Supplemented Zebrafish
Source: Biomolecules. 2023 Jan 9;13(1):136. doi: 10.3390/biom13010136 (PMC9855622; doi:10.3390/biom13010136)
Supplement: Supplementary file 1 [file biomolecules-13-00136-s001.zip › biomolecules-2040484-supplementary.pdf]

Supplemental Figure S1

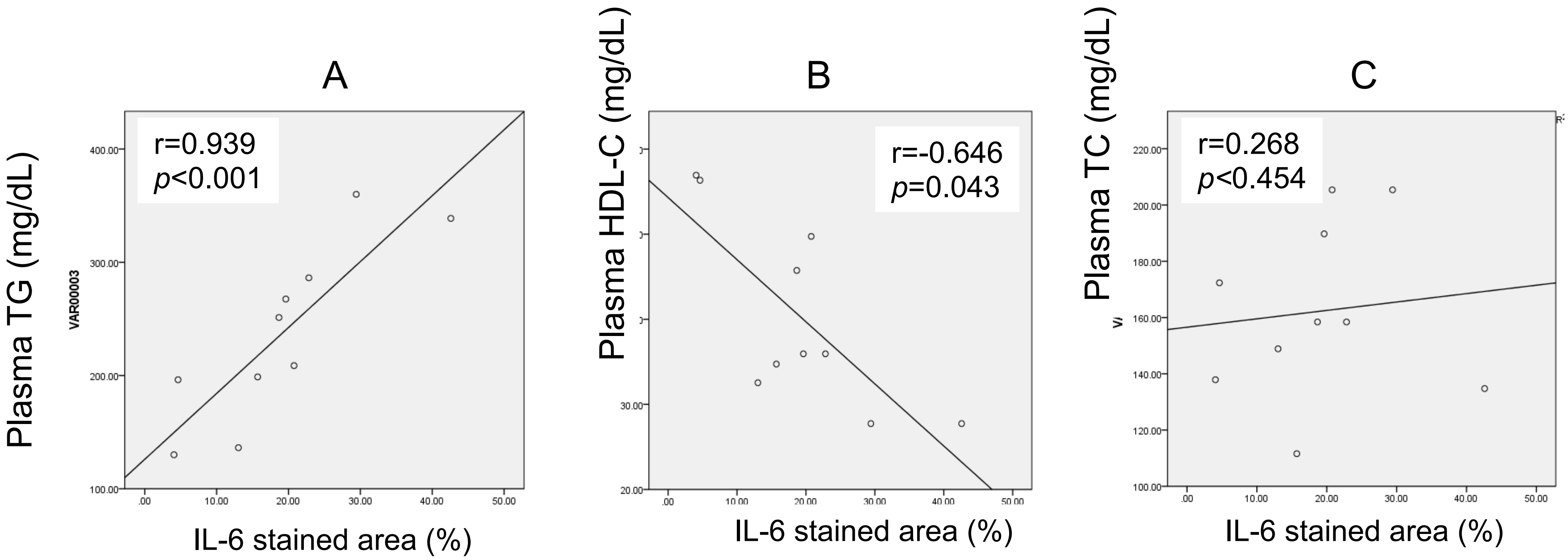

Supplemental Figure S1. Spearman correlation analysis of blood lipid level and IL-6 stained area.
A. Positive correlations was detected in plasma TG and IL-6 area.
B. Negative correlation was detected in plasma HDL-C and IL-6 area.
C. No correlation was detected in plasma TC and IL-6 area.
